# Supplementary material for: Use of E-Health in Dutch General Practice during the COVID-19 Pandemic
Source: Int J Environ Res Public Health. 2021 Nov 26;18(23):12479. doi: 10.3390/ijerph182312479 (PMC8656482; doi:10.3390/ijerph182312479)
Supplement: Supplementary file 1 [file ijerph-18-12479-s001.zip › ijerph-1411082-supplementary.pdf]

File S1. Web survey e-health questions (April 2020)

1. **What is your position within this general practice?**
   - ☐ General practice owner / GP registrar of this practice
   - ☐ Salaried general practitioner in this practice
   - ☐ Locum in this practice
   - ☐ Doctor's assistant / GP assistant
   - ☐ Practice nurse
   - ☐ Nursing specialist or physician assistant in this practice
   - ☐ Practice manager within this practice
   - ☐ Other, namely:________

2. **Have you used (new) e-health applications in your practice because of the COVID-19 pandemic or have you started using existing applications more often? And do you think you will continue to use them more intensively? These can be, for example, electronic patient contact by means of e-consultation or video consultation, or electronic peer contact by means of teleconsultation.**
   - ☐ No, I have not used new e-health applications nor I have started using existing e-health applications more often.
   - ☐ Yes, namely:

	We already used this applications, but started using it more intensively	We started using the following applications for the first time	We believe we will continue to use this application more intensively after the pandemic	Does not apply
E-consultation	☐	☐	☐	☐
Online ordering of repeat prescriptions	☐	☐	☐	☐
Video consultation with patients	☐	☐	☐	☐
Teleconsultation (electronic peer contact)	☐	☐	☐	☐
Telemonitoring (healthcare provider monitors online what the patient is measuring at home)	☐	☐	☐	☐
Other, namely:_____	☐	☐	☐	☐

File S2. Web survey e-health questions (July 2020)

3. **What is your position within this general practice?**
   - ☐ General practice owner / GP registrar of this practice
   - ☐ Salaried general practitioner in this practice
   - ☐ Locum in this practice
   - ☐ Doctor's assistant / GP assistant
   - ☐ Practice nurse
   - ☐ Nursing specialist or physician assistant in this practice
   - ☐ Practice manager within this practice
   - ☐ Other, namely:________

4. **Which e-health applications does your practice use at the moment? You may select multiple answers.**
   - ☐ E-consultation
   - ☐ Request repeat prescriptions online
   - ☐ Video consultation with patients
   - ☐ Teleconsultation (electronic peer contact)
   - ☐ Telemonitoring (healthcare provider monitors online what the patient measures at home)
   - ☐ None of the above e-health applications
   - ☐ Other e-health applications, namely:_________

5. **If you selected 'None of the above e-health applications' can you explain your answer? How did you organize 'remote healthcare' differently in your practice during the COVID-19 pandemic?**

6. **Which of the following e-health applications have you continued to use more intensive in your practice, now that the first wave of the COVID-19 pandemic is over? And which of the following e-health applications do you think you will continue to use more intensively in the future? You may select multiple answers.**

	We have started using more intensively	We think we will continue to use more intensively	We have not started using more intensively or we do not think we will continue to use them more intensively
E-consultation	☐	☐	☐
Online ordering of repeat prescriptions	☐	☐	☐
Video consultation with patients	☐	☐	☐
Teleconsultation	☐	☐	☐
Telemonitoring	☐	☐	☐
Other, namely:_____	☐	☐	☐

Table S1. Respondent's position within the general practice (April 2020 and July 2020)

Position	April 2020 (n=1,598)*	July 2020 (n=843)*
General practice owner	85%	76%
Salaried/employed general practitioner	2%	3%
General practitioner locum	1%	1%
General practitioner assistant	4%	6%
Practice nurse and/or nurse practitioner	1%	1%
Nursing specialist or physician assistant	0%	0%
Practice manager	6%	12%
Other position	1%	1%

*Response numbers are based on question 1 from File S1 and question 3 from File S2, which were the first questions of the complete surveys in April and July 2020.

Table S2. Differences in practice types between general practices in practice sample and population (April 2020 and July 2020).

Practice type	Sample April (n=1,427)	Sample July (n=735)	Population (n=4,911)*
Solo	20.3%	20.2%	24.0%
Duo	45.4%	41.4%	43.9%
Group	34.4%	38.4%	32.1%

*based on the 'Healthcare Professionals Registries' [8]

Table S3. Percentage of practices indicating intensified use of existing e-health applications because of the COVID-19 pandemic, specified by e-health application (mean ± standard deviation), April 2020.

E-health application	Solo practice (n=211)	Duo practice (n=480)	Group practice (n=383)	p-Value
E-consultation	65% ± 0.477	73% ± 0.443	77% ± 0.421	0.009*
Online ordering of repeat prescriptions	66% ± 0.475	70% ± 0.459	69% ± 0.464	0.561
Video consultation	13% ± 0.340	10% ± 0.300	12% ± 0.326	0.406
Teleconsultation	46% ± 0.500	53% ± 0.500	55% ± 0.498	0.095
Telemonitoring	15% ± 0.360	12% ± 0.329	14% ± 0.346	0.567
Other	5% ± 0.213	6% ± 0.235	6% ± 0.238	0.801

One-way ANOVA was used to test differences in the mean use of specific e-health applications between the three practice types. * Indicates a significant difference ($p < 0.05$) between the mean values of the three practice types.

Table S4. Percentage of practices indicating intensified use of existing e-health applications because of the COVID-19 pandemic, specified by e-health application (mean ± standard deviation), July 2020.

E-health application	Solo practice (n=11-123)	Duo practice (n=15-266)	Group practice (n=27-247)	p-Value
E-consultation	64% ± 0.481	72% ± 0.449	69% ± 0.462	0.299
Online ordering of repeat prescriptions	42% ± 0.496	39% ± 0.488	40% ± 0.491	0.814
Video consultation	49% ± 0.504	62% ± 0.487	61% ± 0.490	0.197
Teleconsultation	37% ± 0.486	43% ± 0.496	44% ± 0.497	0.569
Telemonitoring	33% ± 0.492	53% ± 0.506	54% ± 0.505	0.442
Other	45% ± 0.522	47% ± 0.516	67% ± 0.480	0.334

One-way ANOVA was used to test differences in the mean use of specific e-health applications between the three practice types. * Indicates a significant difference ($p < 0.05$) between the mean values of the three practice types. The amount of practices (n) that answered this question in July differed, depending on how many practices were using the specific e-health applications.

Table S5. Percentage of practices indicating they will continue the intensified use of an e-health application after the COVID-19 pandemic, specified by e-health application (mean ± standard deviation), April 2020.

E-health application	Solo practice (n=211)	Duo practice (n=480)	Group practice (n=383)	p-Value
E-consultation	26% ± 0.440	25% ± 0.433	28% ± 0.448	0.674
Online ordering of repeat prescriptions	14% ± 0.350	13% ± 0.340	15% ± 0.354	0.858
Video consultation	23% ± 0.423	26% ± 0.439	32% ± 0.468	0.039*
Teleconsultation	17% ± 0.377	15% ± 0.353	19% ± 0.393	0.212
Telemonitoring	12% ± 0.329	5% ± 0.218	7% ± 0.256	0.003*
Other	3% ± 0.167	2% ± 0.150	4% ± 0.194	0.367

One-way ANOVA was used to test differences in the mean use of specific e-health applications between the three practice types. * Indicates a significant difference ($p < 0.05$) between the mean values of the three practice types.

Table S6. Percentage of practices indicating they will continue the intensified use of an e-health application after the COVID-19 pandemic, specified by e-health application (mean ± standard deviation), July 2020.

E-health application	Solo practice (n=11-123)	Duo practice (n=15-266)	Group practice (n=27-247)	p-Value
E-consultation	41% ± 0.495	38% ± 0.487	36% ± 0.480	0.567
Online ordering of repeat prescriptions	31% ± 0.463	30% ± 0.459	20% ± 0.400	0.015*
Video consultation	42% ± 0.497	38% ± 0.487	35% ± 0.479	0.646
Teleconsultation	33% ± 0.473	38% ± 0.486	31% ± 0.464	0.349
Telemonitoring	58% ± 0.515	39% ± 0.494	46% ± 0.505	0.502
Other	64% ± 0.505	47% ± 0.516	41% ± 0.501	0.454

One-way ANOVA was used to test differences in the mean use of specific e-health applications between the three practice types. * Indicates a significant difference ($p < 0.05$) between the mean values of the three practice types. The amount of practices (n) that answered this question in July differed, depending on how many practices were using the specific e-health applications.
